# Digital Twins in the Practice of High-Energy Physics Experiments: A Gas System for the Multipurpose Detector

**DOI:** 10.3390/s22020678

**Published:** 2022-01-16

**Authors:** Patryk Chaber, Paweł D. Domański, Daniel Dąbrowski, Maciej Ławryńczuk, Robert Nebeluk, Sebastian Plamowski, Krzysztof Zarzycki

**Affiliations:** 1 Institute of Control and Computation Engineering, Faculty of Electronics and Information Technology, Warsaw University of Technology, ul. Nowowiejska 15/19, 00-665 Warsaw, Poland; Patryk.Chaber@pw.edu.pl (P.C.); Pawel.Domanski@pw.edu.pl (P.D.D.); Robert.Nebeluk@pw.edu.pl (R.N.); Sebastian.Plamowski@pw.edu.pl (S.P.); Krzysztof.Zarzycki@pw.edu.pl (K.Z.); 2 The Faculty of Physics, Warsaw University of Technology, ul. Koszykowa 75, 00-662 Warsaw, Poland; Daniel.Dabrowski.dokt@pw.edu.pl

**Keywords:** digital twins, Simscape, multipurpose detector, gas system, time-of-flight detector, gas mixing

## Abstract

The digital twins technology delivers a new degree of freedom into system implementation and maintenance practice. Using this approach, a technological system can be efficiently modeled and simulated. Furthermore, such a twin offline system can be efficiently used to investigate real system issues and improvement opportunities, e.g., improvement of the existing control system or development of a new one. This work describes the development of a control system using the digital twins methodology for a gas system delivering a specific mixture of gases to the time-of-flight (ToF) multipurpose detector (MPD) used during high-energy physics experiments in the Joint Institute for Nuclear Research (Dubna, Russia). The gas system digital twin was built using a test stand and further extended into target full-scale installation planned to be built in the near future. Therefore, conducted simulations are used to validate the existing system and to allow validation of the planned new system. Moreover, the gas system digital twin enables testing of new control opportunities, improving the operation of the target gas system.

## 1. Introduction

Digital twins technology is a relatively new notion [1,2]. It brings well-known modeling and simulation technologies to a new level [3]. Digitization in the industry is perceived as an opportunity to achieve higher levels of productivity [4,5]. The standard interpretation and use of data in combination with the digital equivalent of the actual system (existing or planned) open up a new perspective on the design, maintenance, and improvement capabilities of real plants and, ultimately, the optimization of costs and the manufacturing process [6]. The Gartner Group, a leading research and advisory company, identified digital twins as one of the top ten recent strategic technology trends [7,8,9].

According to the digital twin approach, a replica of some physical object is developed and it can be seen as a wide-use simulator [10], as a predictive model, and a tool that enables equipment prognostic and diagnostics [11] by monitoring and remotely detecting, in real time, anomalies that may result from equipment failures. In the case of complex systems, a digital twin can be used as a training system for operators. The digital twin can also be a part of a cybersecurity system enabling infrastructure protection against attack [11,12]. In process control, a digital twin of the real process enables testing and validation of various control strategies and scenarios. It must be emphasized that control system performance strongly depends on the available sensors and actuators. In the literature, numerous processes have been considered using the digital twin methodology: a water cooling system in a power plant [13], an optical measurement system [14], an indoor safety management system [15], a field perception method in aircraft assembly [16], fan-blade reconditioning for aerospace maintenance [17], automatic transportation [18], railway turnouts [19], and smart manufacturing [20,21,22].

Numerous attempts are being made to standardize the digital twins methods—the Institute of Printed Circuits (IPC) organization has announced the release of IPC-2551, an international standard for digital replicas. Standardizing requirements means fulfilling them, and good practices will undoubtedly facilitate their design and increase interoperability. The details of their implementation, however, always depend on the case and particular project requirements. There are several industries, such as manufacturing [23,24,25,26,27], automotive [28,29], healthcare [30,31,32,33], aviation, and terrestrial exploration [34,35], where the digital twins concept has been successfully deployed.

This work presents an application of the digital twins methodology in the area of high-energy physics experiments. The objective of this work is to describe the development of a control system for a gas system (GS) delivering a specific mixture of gases to the time-of-flight (ToF) Multi Purpose Detector (MPD) used during the high-energy physics experiments in the Joint Institute for Nuclear Research (JINR) [36]; it is located in Dubna, Russia [36]. The MPD is an original original technical solution currently being developed in JINR. The ToF detector is used to detect particles through the discrimination between lighter and heavier elementary particles, measuring their time of flight between two system parts. A crucial feature of the system is to keep proper gas conditions (pressure and composition), which fills the space between both plates of the detector. The GS delivers proper gas, and the digital twin aims to replicate the GS. In actuality, the GS exists in a testing stand version with a limited number of detectors. It is used to prove applied technologies. Simultaneously, a full-scale ToF detector is still under construction and does not exist in full scale. Once accomplished, it must be equipped with an efficiently working gas system. This fact ultimately justifies the selection of the digital twins approach. The described gas system relies on multiple sensors. Proper functioning of sensors is necessary for the correct functioning of the system itself and the controllers. The importance of sensors and accuracy of measurements is an essential issue in many advanced monitoring systems, e.g., [37,38,39,40].

This work describes the development of the digital twin of the GS, first for a test system and further for a full-scale target system. It is subject to validation using real process data. Next, the virtual system is equipped with a control system, which assures control of the pressure and gas composition. Digital replica of the GS enables testing and selecting control strategy that can be further implemented into the target control hardware.

The analysis starts with the MPD ToF description in Section 2, followed by the introduction to the possible simulations technologies presented Section 3. Section 4 forms the main contribution and describes the digital twin application for the MPD ToF gas system, consisting of the system description and the results. Section 6 concludes the paper, presenting identified open issues for future investigation.

## 2. Multi Purpose Detector

During the high-energy physics experiments, particles are accelerated and then smashed in the Nuclotron-based Ion Collider fAcility (NICA) collider [41]. As a result, hundreds of new particles are created, flying in all directions. At the point of the interaction, the MPD [42] is located. Its main task is to collect data about hot and dense barionic matter properties by analyzing particles created in such collisions. One of the essential detecting systems of the MPD is ToF, which requires keeping its components in specific atmospheric conditions. Thus, there is a need to keep a proper mixture of gases with constant composition. Simultaneously, the detector’s electronic circuits generate large amounts of heat, which generates an obligatory need for temperature stabilization.

This brief introduction shows that two systems, i.e., GS and thermal system, need to work correctly for proper ToF detector operation. This work focuses on the gas system described after a short presentation of the ToF detector.

### 2.1. Time-of-Flight Detector

**Figure 1:**
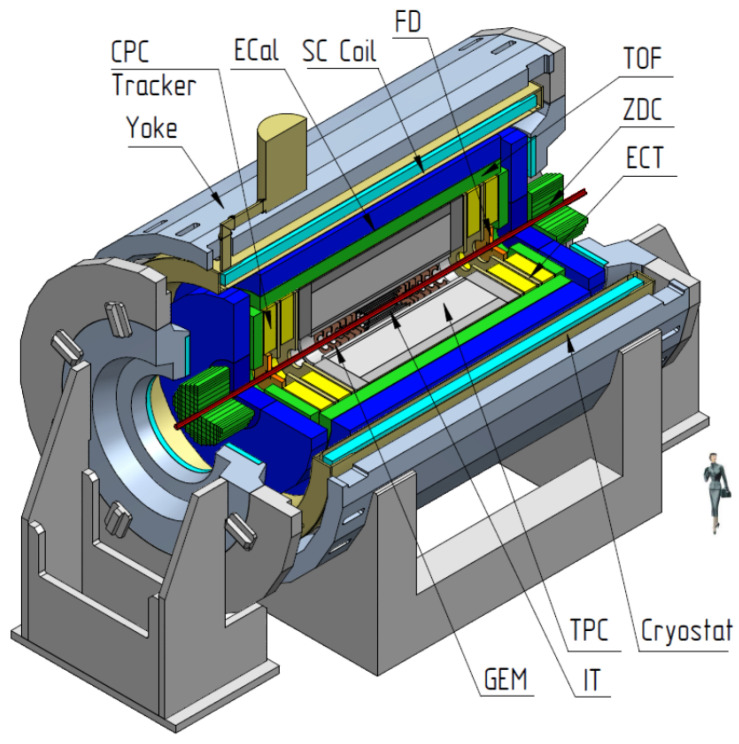
The MPD detector with end doors retracted for access to the inner detector components; the detector includes several different detectors: Time projection chamber (TPC), time of flight (ToF), electromagnetic calorimeter (Ecal), fast-forward detector (FFD), forward Hadron calorimeter (FHCal), and inner tracking system (ITS). Other systems: Superconductor solenoid (SC coil) and magnet yoke, inner detector (IT), straw-tube tracker (ECT), time-projection chamber (TPC), time-of-flight system (TOF), electromagnetic calorimeter (EMC), and zero-degree calorimeter (ZDC).

The entire MPD detector consists of many different sub-detectors performing different forms of particle detection, as shown in Figure 1 [43]. The ToF is only one of its components. It is situated between the main tracking detector, which is the time projection chamber (TPC) and the electromagnetic calorimeter, which measures the momentum of photons and electrons. Its construction resembles a barrel with an internal diameter of approximately 3 m and an external of 3.4 m. Fourteen pairs of modules are placed on a circle with a distance of less than 5 mm.

**Figure 2:**
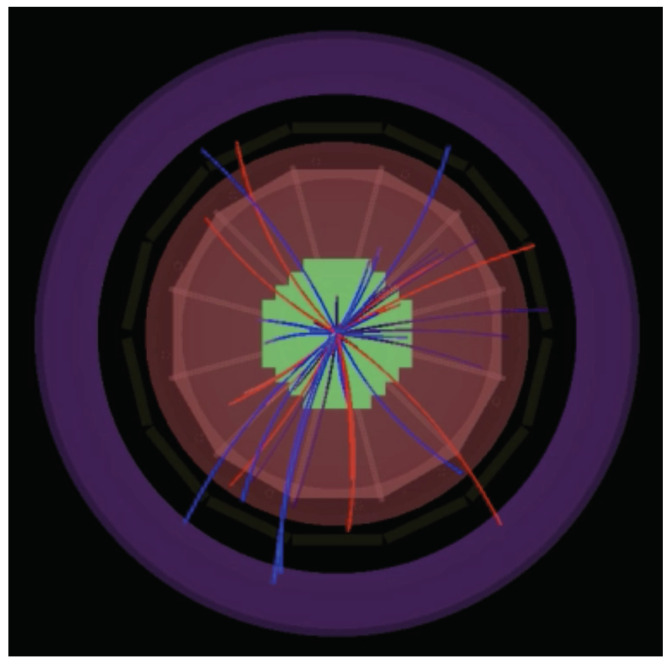
Tracks of the particles created in the simulated heavy ion collision (using UrQMD generator and MPD detector geometry), where negatively charged particles are marked in blue, and positive in red.

The operation of the ToF detector is very simple – it measures newly created particles’ time of flight, from the moment of the collision in the middle of MPD (START signal triggered by the FD detector) until the given particle passes through the detector (STOP signal generated by the ToF). To identify a particle (calculate its mass *m*), the following formula is used:

(1) $$m^{2}=\frac{p^{2}}{c^{2}}\left[{\left(\frac{1}{\beta }\right)}^{2}-1\right]$$

where *p* stands for the momentum, $\beta =\frac{L}{Tc}$ is the velocity of a particle with respect to the speed of light, *L* is the length of the particle’s track, *T* is the measured time of flight, and *c* is the speed of light. During the experiment, three parameters have to be measured: the length of its track (examples of the tracks are shown in Figure 2), momentum (both measured by TPC detector), and time of flight from interaction point to the ToF detector.

**Figure 3:**
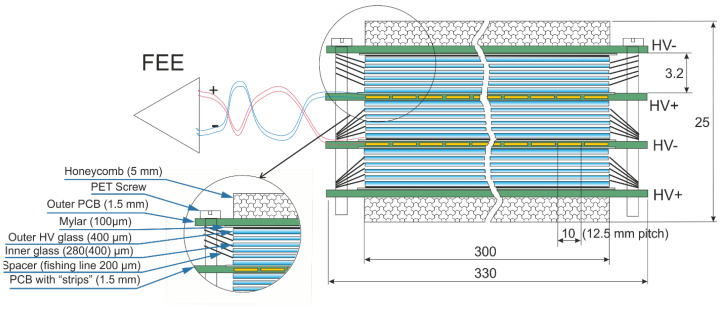
Scheme of the triple-stack MRPC for ToF MPD.

To register particles flying through the ToF detector, multigap resistive plate chambers (MRPC) are used. They are shown in Figure 3. There are ten of them in each ToF module. The MRPC consists of a stack of plates separated from each other by separators of equal size to form gas gaps. On the outer surface, there is a high-voltage coating that generates an electric field. As the particles pass through the detector, they ionize the gas inside. The flow of electrons and ions generates the voltage on the inner plates through the gas gap [44]. It is recorded as an impulse transferred to the second chamber which is the pre-amplifier chamber. The recorded signal combined with the knowledge of the collision moment observed in the FD detector allows determination of the particle time of flight [45].

For proper operation, MPRCs must be closed in a tight chamber filled with a specific gas mixture. Parameters of the gas environment have a major influence on its registration possibilities. Ensuring the right and stable gas environment is crucial for proper functioning of the ToF detector. Therefore, so much attention is paid to the implementation of the gas system.

### 2.2. Gas System

The purpose of the gas system is to ensure constant overpressure and gas composition in the detector chambers. The gas system must meet the following gas technical requirements:

- To keep the overpressure inside the detector chambers of 3–5 mbar.
- To keep the gas flow through each detector chamber of the order 200–300 ccm.
- To maintain dynamically a defined gas mixture composition: $90\%C_{2}H_{2}F_{4}+5\%{\mathrm{SF}}_{6}+5\%{i-C}_{4}H_{10}$.
- To limit H_2_O and O_2_ pollution below 1000 ppm.
- To control gas recirculation in a closed loop.

Keeping slight overpressure reduces the ingress of air into the detector chambers, but the mechanical structure is only able to withstand 10 mbar overpressure. The exact value of the pressure, however, does not have a significant impact on the detection capabilities of the ToF. Gas flow through detector modules allows to maintain mixture homogeneity and prevents $H_{2}O$ and $O_{2}$ accumulation inside of the detector chambers [46]. Those impurities come from residual leakages, materials out-gassing, and diffusion through silicone sealant. In particular, the water vapors contamination may significantly affect detector performance by reducing efficiency, signal rates, and rising currents [47]. Therefore, the gas leaving the detector chambers is continuously analyzed and purified. As soon as the water content begins to rise, indicating that the purifier column is saturated, it is replaced.

Gas mixture composition and, hence, its properties are crucial for the performance of the ToF detector. Efficiency and gas gain directly depend on the effective Townsend coefficient, while ToF time resolution is associated with the gas electron drift velocity and effective Townsend coefficient. Simulations show that addition of the i-$C_{4}H_{10}$ in the $C_{2}H_{2}F_{4}$ improves time resolution [48]. On the other hand, working with the explosive isobutane and isobutane-rich mixture brings with it additional handling difficulties. Moreover, the working voltage range for such a mixture is quite short; a small fraction of ${\mathrm{SF}}_{6}$ may be added to extend working voltage range and simplify work with such gas [49]. The addition of ${\mathrm{SF}}_{6}$, which is strongly electronegative gas, also has the benefit of suppressing the transition to streamers inside of the MRPCs, which could damage the detector. Unfortunately, ${\mathrm{SF}}_{6}$ also suppress the development of the electron avalanche in it (signal), so its content must not be large and it is necessary to apply a higher operating voltage to reach MRPC efficiency plateau [50]. In conclusion, the research shows that the optimal mixture ratio is $90\%C_{2}H_{2}F_{4}+5\%{\mathrm{SF}}_{6}+5\%{i-C}_{4}H_{10}$ [51].

Due to the abovementioned effects, maintaining correct mixture proportions is essential, and the implemented gas system fulfills this task perfectly. Any deviations would be noticed immediately during the testing procedure, for example, through increased currents or streamers production. Multiple ToF chambers are always tested simultaneously (usually eight of them), and only malfunction of most or all of them would suggest problems with the gas environment. Such a situation did not take place yet. However, incorrect parameters of individual detectors are noticed rarely. As it turned out later, it has been always caused by a blocked or bent pipe supplying gas to a given chamber, resulting in the lack of gas flow.

**Figure 4:**
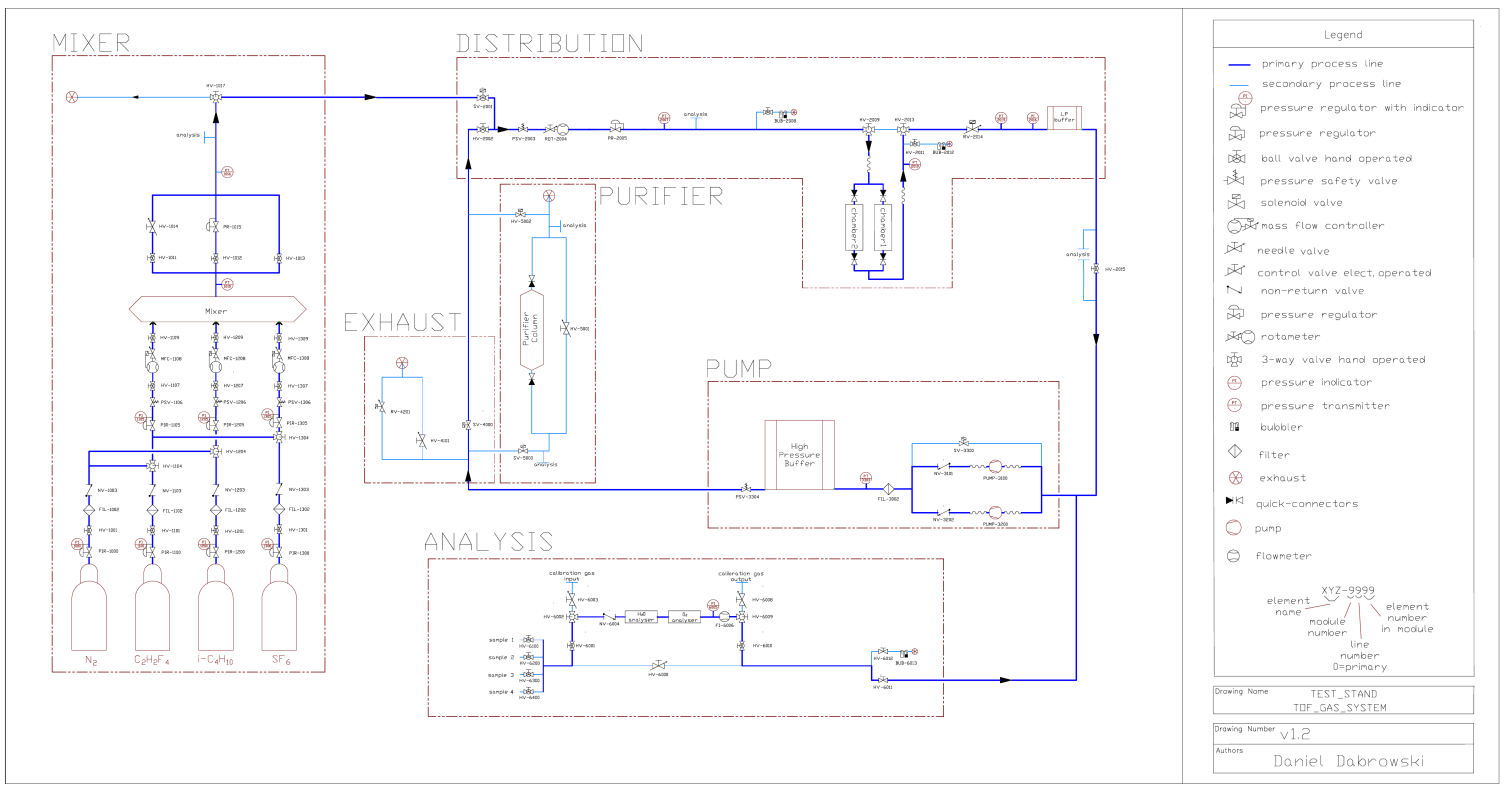
Gas system P&ID drawing—pilot version.

The individual components of the gas system are described below, presenting the existing test system in a pilot version. Figure 4 shows the test gas system in the form of the piping and Instrumentation Diagram (P&ID) drawing.

#### 2.2.1. Mixer

The main task of the mixer is to dynamically prepare a gas mixture with a given composition and deliver it to the other parts of the system under a given pressure. Three mass flow controllers from MKS Instruments constitute the system’s core, limiting maximum available flows at 1000 sccm, 100 sccm, and 50 sccm, respectively. After scaling by the gas conversion factor for the used gases (0.27–0.35), the maximum available flow, 300 ccm, of the mixture is obtained. This flow is sufficient to test one or two detector modules at the pilot system.

Similarly to the rest of the system, the mixer can operate in three main modes: purge, fill, run, and manual (direct). In the purge mode, the HV-1104, HV-1204, and HV-1304 three-way valves are set to allow nitrogen to flow through all lines. In the fill and run modes, they are put through the target gas mixture. In purge and fill modes, the control aims at keeping an overpressure of 250 mbar inside the output tank of the mixer (3.8 L capacity) and stable proportions of the gas mixture, trying to keep the gas flowing through the mass flow controllers. A proportional-integral-derivative controller (PID) control loop is used to maintain the pressure setpoint. The PID controller calculates the required total gas flow to maintain the set pressure value. In this mode, the impact of potential disturbances is negligible, except in critical cases when someone deliberately opens the mixer tank, or the gas in the cylinder runs out. Moreover, the pumps may force a flow greater than the mixer module can deliver. Then, the overpressure in the mixer tank drops to about 10 mbar, despite the maximum opening of the mass flow controllers [52]. In the run mode, where the closed recirculation circuit is activated, the mixer ought to work continuously, maintaining the flow:

- Equal to the sum of leaks throughout the system increased by some amount necessary to maintain proper pressure inside the system, taking into account changes in atmospheric pressure.
- Additionally increased by an additional small value to refresh the gas.

#### 2.2.2. Distribution

In the prototype layout, the distribution module is straightforward and practically free of automation elements. It is purely mechanical and is responsible for the delivery of the gas to the ToF detector elements. In the target construction, a functionality of leakage detection will be added due to the much larger surfaces and volumes.

#### 2.2.3. Recirculation

Pump, exhaust and purifier modules are merged into the recirculation, as there is no need to separate them in a pilot version. The task of the recirculation module in purge and fill modes is primarily to maintain the pressure at the output of the detectors setpoint, thus ensuring gas flow through its modules. The PID control loop performs this function. The essential disturbance is the change in atmospheric pressure, which causes a significant difference in the operation of the pump. Another necessary disturbance can be a rupture of the diaphragm in the running pump, which is rare but still possible.

#### 2.2.4. Gas Supply

Currently, the gas supply module is only equipped with sensors to monitor gas consumption in cylinders. It is planned to add an electropneumatic valve, allowing for automatic switching between two freon cylinders.

#### 2.2.5. Purifier

A disposable purifier column is used as a purifier, which is replaced when saturated.

#### 2.2.6. Analysis

The manual valve is used to collect the gas sample for analysis. The measured water and oxygen content values are only shown to the operator on the operator’s screen.

### 2.3. Target Gas System Configuration

**Figure A1:**
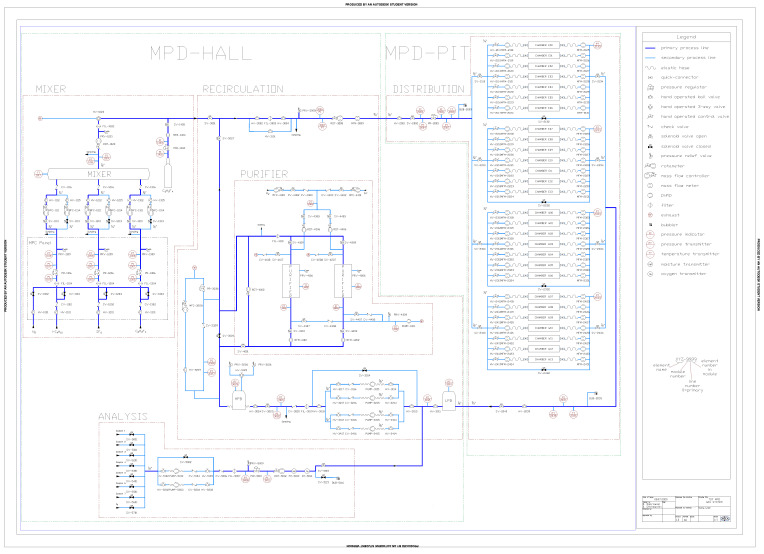
P&ID diagram of the target system layout.

The designed target form of the gas system will be larger. Its structure in the form of a P&ID drawing is shown in Figure A1. The detector itself will be divided into four sectors with added solenoid valves, which will allow the remote shutdown of a given sector once a leakage is detected. The basis for leak detection of the detector’s chambers will be mass flow meters at the input and output of each of the 28 chambers. Each sector will also be equipped with a pressure transmitter.

The purifier will be separated from the recirculation, and additional measuring and actuating devices will also be available, such as the mainline total flow meter or the exhaust flow controller. The system will also be equipped with four pumps, two of which will work simultaneously.

The gas supply system will be equipped with automatic switching between the cylinders. It will also be necessary to add sensors, shut-off valves, and other safeguards related to the storage of isobutane and the relevant procedures implemented in the control software. The purifier column is planned to be exchanged with regenerative columns. Gas analysis will be activated from the program level, resulting in the opening of the corresponding solenoid valve. It is planned to implement three options:

- Manual switching from the user interface.
- Continuous sample analysis from one place.
- Loop switching between all/selected samples.

Additionally, the analysis results will be able to influence the flow through the purification module (purifier) as the gas can be directed entirely to the purification, partially, or in case of low contamination content, it may omit the purifier.

## 3. Simulation Technologies

The simulator architecture was designed to ensure separation of the control layer (SCADA system, i.e., the supervisory control and data acquisition system) from the module simulating the operation of the object. Such a separation allows to develop the control system and the process simulator independently. An additional advantage of the separation is the possibility to apply the prepared control system to the real object.

As the main software for process simulation, the MATLAB software with Simulink and Simscape library is used. The main advantages of this toolset are the ability to simulate dynamic states and the flexibility of software development. The environment is extensively used to model the multidomain systems having complex interactions, such as mechanical and electrical systems [53,54], as well as for modeling the distribution of pressures, temperatures, and flows in gas and liquid systems [55]. In this project, simulation is designed with a connection to programmable logic controllers (PLCs) in mind.

The Simscape library allows to define gas used in the system and its structure with the help of predefined blocks, e.g., pipes, valves, and mass flow controllers. It allows to mirror the structure from P&ID into the simulation environment, which helps keep up to date with changes in the pilot version of the GS. This dependency can also be reversed; thus, each change, e.g., in the control structure of the simulated GS, can be reflected in the control structure of the final version of the GS.

The Simulink software allows to define a set of differential equations describing physical relations between real-life variables. In this case, a subset of those variables and relations that focuses on gases is considered. Therefore, only pressures, flows (of volume or mass), and temperatures are measured, corresponding to the measurements planned in the P&ID. Nevertheless, Simulink allows to use more than one domain, e.g., introducing heat convection between gas system elements, although it is not yet utilized, as there is no temperature sensor in the pilot GS.

Input signals for the simulation correspond to the signals generated by the PLC, whereas measurements of flow, temperature, or pressure are represented as outputs. This allows to encapsulate simulation in a block that can be seamlessly replaced with the real GS. This design allows for both hardware-in-the-loop testing and pure simulated evaluation of the system. Therefore, thorough simulated testing can be performed without the need to reconfigure hardware or reprogram PLC. When changes are verified and approved, they can be implemented in the PLC connected to the simulated GS. After further testing and verification, the simulated GS can be substituted with the real one, which will finalize full implementation.

The connection between the simulated environment and PLC is conducted with the use of OPC UA protocol. This protocol allows for reading and writing registers of the PLC, thus allowing data transfer between the Simulink implementation of the simulated process and the SCADA implemented on the PLC.

## 4. Digital Twin Application for MPD ToF Gas System

The digital twins approach is used to address the issue of gas control system design and validation. The system is validated from two perspectives. First of all, the digital twin of the test model is used as an initial verification platform before system expansion into the full scale. Secondly, a digital simulation platform enables to validate hardware assumptions about control hardware, i.e., PLC and SCADA system.

### 4.1. Simscape Process Model

**Figure 5:**
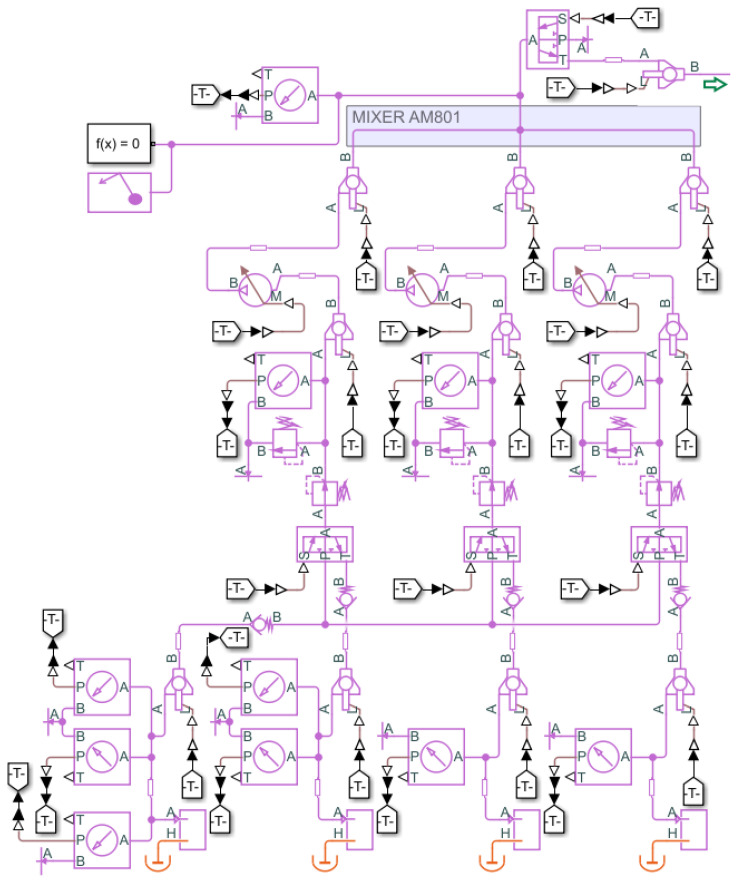
The simulation model of the mixer subsystem.

**Figure 6:**
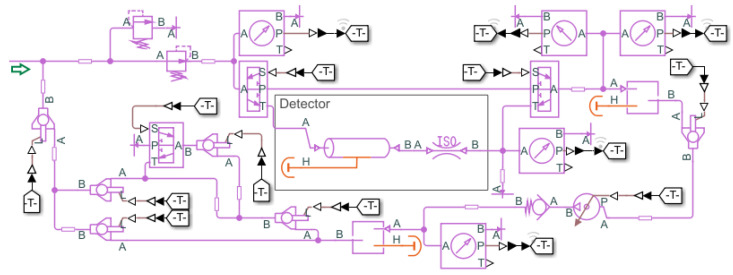
The simulation model of the recirculation subsystem.

A simulation model has been built with the use of Simscape. It consists of two main gas cycles. The mixer is responsible for gas preparation, while the gas circulation loop addresses its regular operation. Figure 5 presents the gas mixing Simscape model. Figure 6 shows the gas recirculation system layout in the Simscape simulation environment. The implemented system has been further validated against pilot system historical data to confirm assumptions and simplifications (see Section 5).

### 4.2. Control System

**Figure 7:**
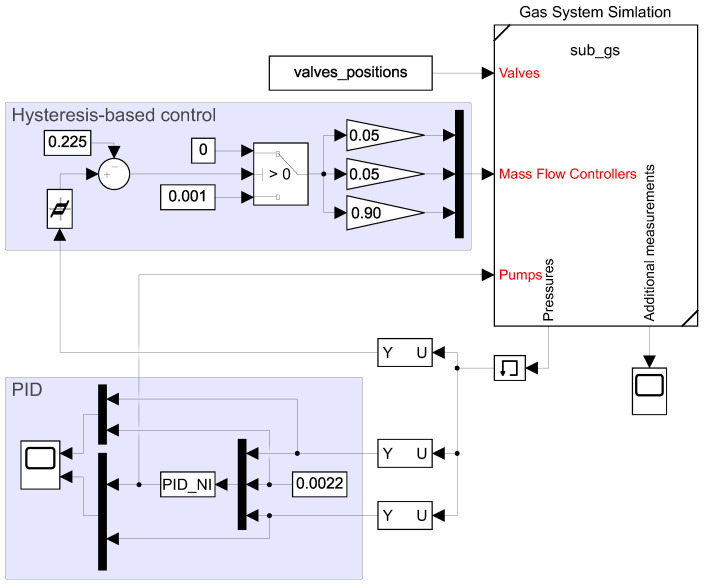
The control structure of the gas system used for recreation of the results obtained using the pilot gas system.

The basic control structure for the gas system is presented in Figure 7. This structure corresponds to the structure already used in the test pilot gas system. It includes a hysteresis-based control for the mixer subsystem and a PID controller to control the pump efficiency that utilizes the same parameters as the pilot system. The PID algorithm is compliant with an implementation from the National Instruments controller being used in the pilot system.

### 4.3. SCADA System

**Figure 8:**
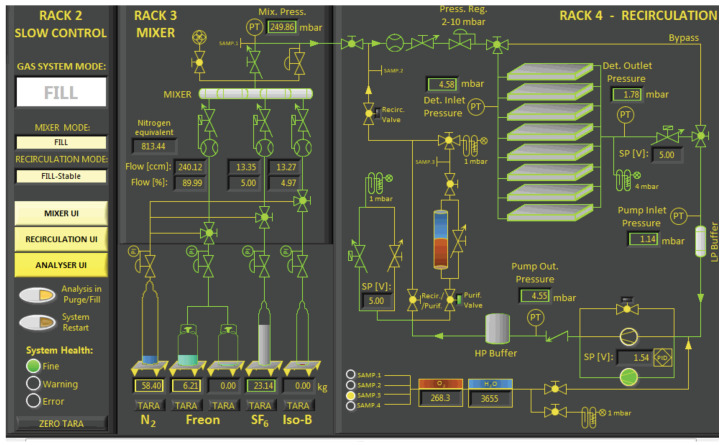
The existing gas system operators screen—the pilot version.

In the future, the real GS will be controlled via the programmable logic controllers (PLC). The human–machine interface (HMI) and supervisory control and data acquisition (SCADA) system are essential for GS to function properly. An existing operators screen (before the project) visualizing system operation is sketched in Figure 8.

**Figure 9:**
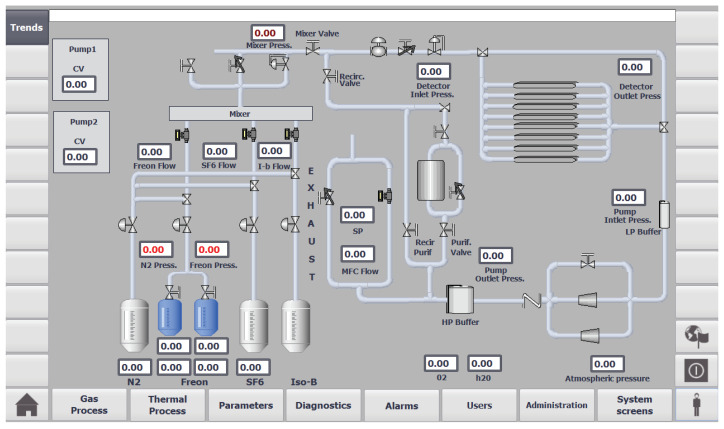
The digital twin SCADA gas process main operators screen.

An attempt has been made to take those requirements into account with the digital twin GS system. A virtual PLC has been deployed by using Siemens PLCSIM Advanced software. The PLC is able to communicate with the simulated GS via an OPC server. A WinCC SCADA system has also been developed with a simple database contacting data about important signals in the system and basic HMI. One of the SCADA screens is presented in Figure 9. This screen shows values of various flow and pressure measurements of the gas system operation. Users can also send control values to pumps and valves in the GS. If the current value of a specific variable is above or below a certain threshold, then the numeric value is highlighted in red.

**Figure 10:**
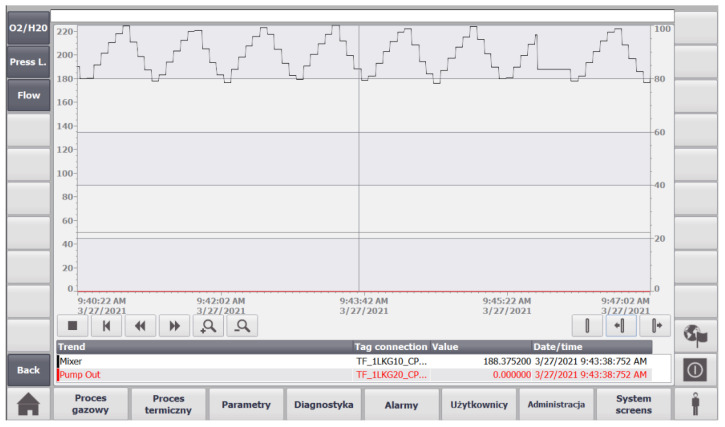
The digital twin SCADA gas process diagnostics screen.

**Figure 11:**
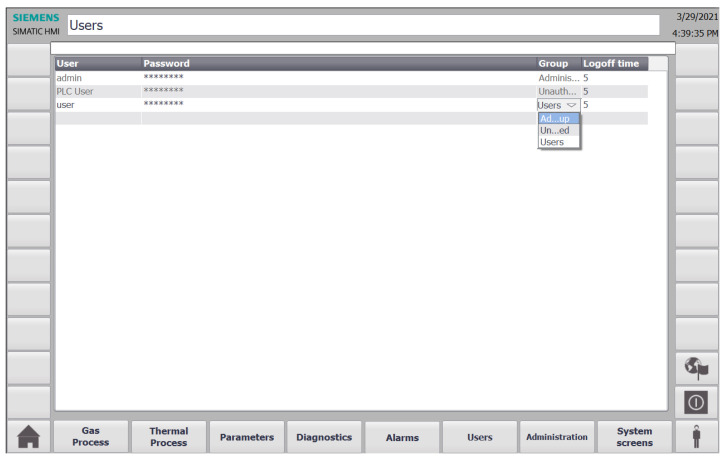
The digital twin SCADA gas process users screen.

**Figure 12:**
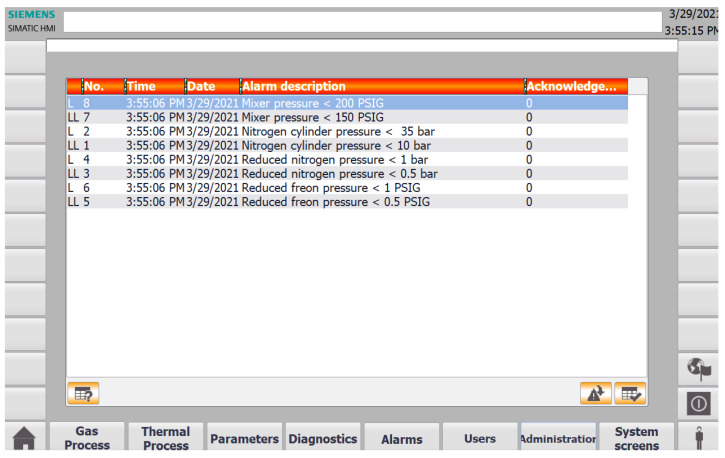
The digital twin SCADA gas process alarms screen.

There are several buttons on the bottom of the screen shown in Figure 9 which can be used to navigate through the HMI screens and experience the full functionality of the SCADA system, such as viewing detailed information about alarms, users, and diagnostics. In the left corner of the diagnostics screen, there is also a button that can be used to show the user historical and current values of specific variables graphically in the form of trends, as shown in Figure 10. In addition, at the bottom on the right-hand side, there is a button for the user to enter their credentials to restrict access for specific parts of the system, as depicted in Figure 11. It allows to differentiate whether the user can clear system alarms, or only acknowledge them; an example alarm screen is shown in Figure 12.

## 5. Model Validation and Simulation Results

**Figure 13:**
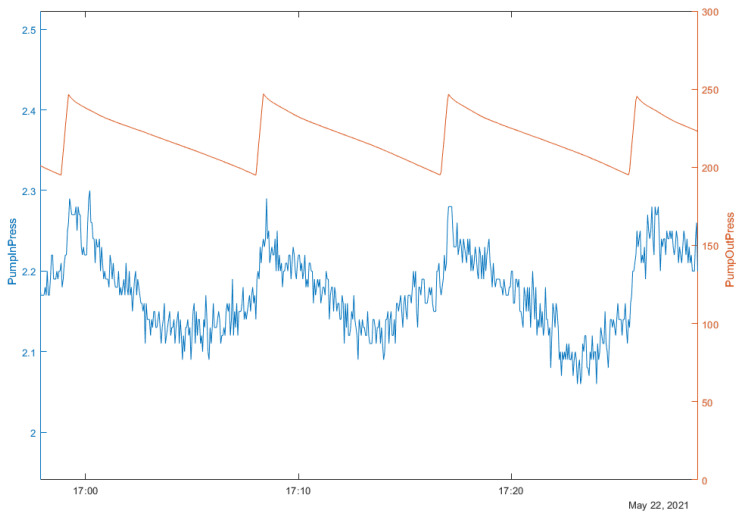
Pump inlet pressure (mbar) and pump outlet pressure (mbar), as measured in the pilot system.

To verify and tune the simulated system, an experiment on the pilot version of GS was performed. A significant leak was introduced to the GS while measuring the response of the pump’s controller. Thus, inlet pressure and outlet pressure of the pump were measured (see Figure 13), in which the leakage causes the pump’s outlet pressure to drop constantly, to the point where the mixer controller activates and introduces a fresh portion of gas, increasing outlet pressure of the pump. This also influences the inlet pressure, as shown in Figure 13.

As is visible in Figure 13, the expected scenario is that pump outlet pressure should be in the range of 200 mbar to 250 mbar, while pump inlet pressure should oscillate around 2.2 mbar. Compared to the results from simulation shown in Figure 16, it is visible that the desired range of controlled signal doubled in the simulated environment. Nevertheless, this level of precision is fully satisfying for further research and work on the control structure. It will also be the subject of further fine-tuning in the later stage of the research. The reference control structure used in the pilot gas system, used to control pump inlet pressure with the efficiency of the pump, was designed to be a simple PI controller with parameters of gain equal to −0.5 and integral constant equal to 0.15.

The complete PID controller was implemented as an S-function Simulink block. S-functions provide possibilities to write easy and advanced algorithms as well as integrate them with other blocks in a Simulink project. Thanks to a set of callback methods that perform the required tasks at each simulation stage, it is possible.

Simulated results of this control structure are presented in Figure 16. The obtained results suggest that there are minor discrepancies between model and real-world data, although due to other errors in modeling and further development of the gas system, those inaccuracies are negligible. The proposed control structures address problems visible in the simulated environment, even if some cannot be easily observed in the trends from the pilot gas system due to the measurement noise. It is worth noting that the size of the leakage is not measured. Thus, the rate at which the gas leaks has not been precisely tuned, as it is only used to lower the period between the mixer’s activation.

**Figure 14:**
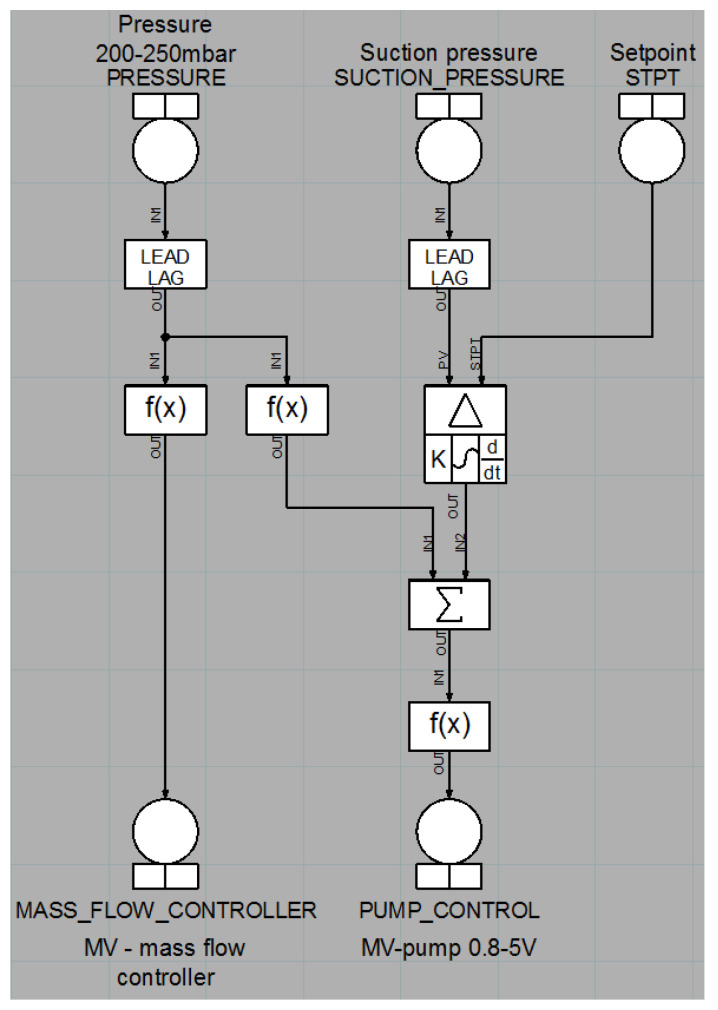
The base structure with static feed-forward module.

The first considered modification of the control structure is to use feed-forward, i.e., the control signal calculated by the PID controller is summed with an additional signal multiplied by a constant value. As this additional signal, TF1LKG20CP204 was selected, i.e., the pressure in the high-pressure part of the recirculation subsystem. This kind of control is classified as a static feed-forward; the used structure is presented in Figure 14.

**Figure 15:**
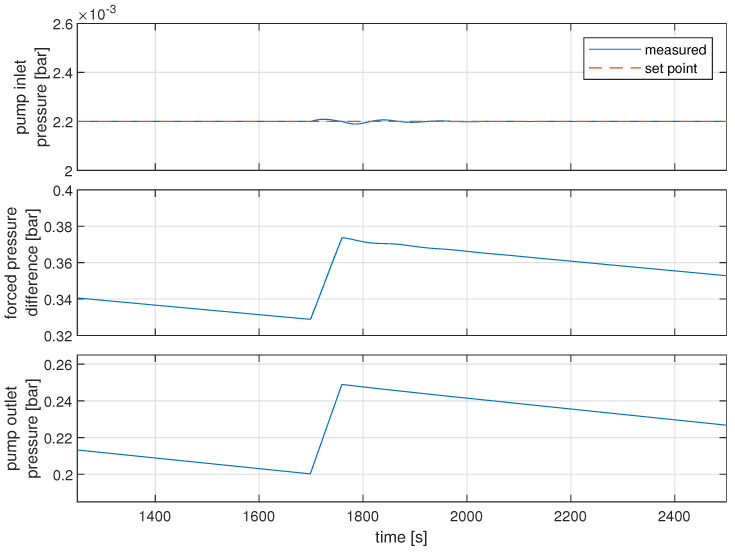
Pump inlet pressure (bar) (**top**, blue solid line) and pump inlet pressure set point (bar) (**top**, red dashed line); pressure difference generated by the pump (bar) (**middle**) with pump outlet pressure (bar). (**Bottom**) PID with feed-forward.

**Figure 16:**
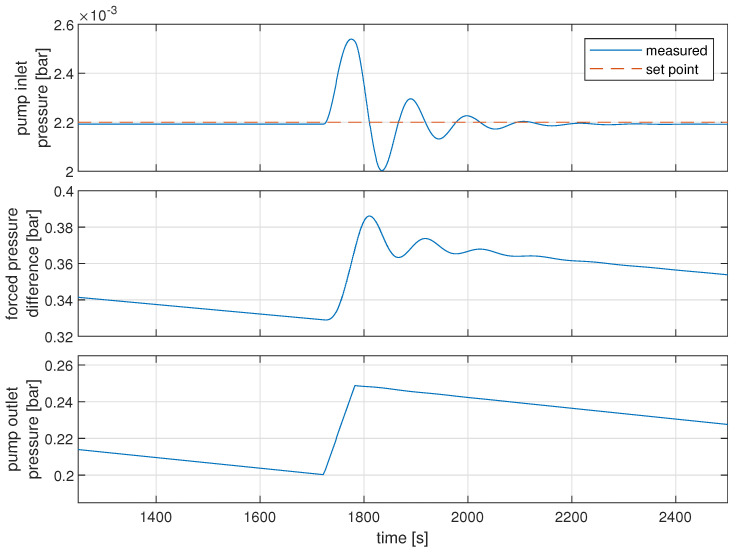
Pump inlet pressure (bar) (**top**, blue solid line) and pump inlet pressure set point (bar) (**top**, red dashed line); pressure difference generated by the pump (bar) (**middle**) with pump outlet pressure (bar). (**Bottom**) reference control structure.

The considered control structure works very well when the speed of disturbance and dynamics of the control signal are similar. In the first approach, a constant value is used as the characteristic F(x). The gain is of the value 0.9; it was selected during performed experiments. The results obtained using this structure are presented in Figure 15. It is visible that the quality of control is greatly increased compared with the case when the feed-forward action is not used (Figure 16), and the controlled variable stays in the range of 2.189 mbar to 2.208 mbar, although this result can be further improved by fine-tuning the feed-forward gain. The considered control structure may be sensitive to changes in operating points.

Above attached simulations show initially obtained agreements between real gas system installation and its digital twin. Presented data show scenarios of the system’s regular operation. The installation’s very slow dynamics (counted in days) should also be highlighted. Realization of any plant experiments is very tedious and labor-intensive. Any experiment disturbing regular operation requires justification and supervisors’ approvals, as it disables regular operation of the installation of the physical experiment. Other operational phases, such as startup or shutdown procedures, of the gas system are performed manually by operators. Therefore, it is impossible to exactly repeat those changes in the digital twin. Nonetheless, further digital twin validation will be performed, as the installation is operational and disturbances appear. Especially as those situations when disturbances occur, though infrequent, are the most valuable for proper digital twin tuning.

### Impact on Real Gas System

The proposed digital twin of the gas system has a significant impact on the real gas system, as it increases the current safety and safety of its development. Increased safety is guaranteed by using the digital twin as a pool of redundant sensors. In case of a failure, a comparison of real signals with simulated ones allows fast detection of errors in the system. On the other hand, any further development can be performed firstly on the simulated GS, allowing to introduce significant changes in the structure of the GS without the risk of it being damaged or even destroyed.

Of course, the accuracy of the simulator is not without significance. As we already mentioned, the bigger facility system does not yet exist, so its simulator’s accuracy cannot be examined. However, it is worth noting that the pilot system was built as a scaled-up industrial system. It captures the essence of industrial system behavior. Secondly, it is important to stress that the simulator uses physical parameters of the system’s elements, e.g., lengths and diameters. Such parameters are utilized in the simulator of the pilot process, and high accuracy was obtained; hardly any final tuning of the parameters was necessary. This observation allows us to assume that entering physical values of the parameters corresponding to the real bigger industrial system will allow us to reproduce the properties of the big industrial system very well.

One of the ongoing work objectives is to develop a control structure. It is a well-known fact that an effective control structure must take into account the characteristics of the process represented by its simulator. Even if the simulator does not perfectly imitate the process behavior, it will show the properties of the process, and it will enable us to develop a control structure in an appropriate form. Moreover, when applied to the real process, such a control structure will only require minor tuning. Considering the properties of the simulator developed so far, the authors are convinced that the general structure of the available simulator will be correct, and modifications, if any, will not be significant.

Even if no thorough test can guarantee that the real GS yields the same results as the simulated one, it is almost certain that the system will remain undamaged. Moreover, this simulated GS can be used to perform online optimization of the process operating point. This digital twin is formally an accurate model of the real GS.

## 6. Conclusions and Future Research

This work discusses an application of the digital twins approach in development of the control system for a gas system used in high-energy physics experiments. The existing pilot process is modeled using MATLAB Simulink Simscape environment. Next, it is equipped with a digital twin model representing the existing control system. The obtained simulation results are cross-checked with real-time operational data. Next, the system is extended with a digital twin for a control infrastructure, including controllers and SCADA system. Finally, the whole system is extended into the virtual model of a planned, but not yet existing, full-scale gas system. Control structures are proposed and validated. Therefore, future system realization is made easier with the mitigated project risks.

The digital twins methodology proves its usefulness in the considered application. On the other hand, it is necessary to stress that although the general idea of the considered approach is straightforward, its actual realization requires the use of specific knowledge originating from different research areas, such as data analytics, simulation and modeling, machine learning, process control, and, last but not least, the specific technological knowledge about the real target system. 

## Data Availability

Not applicable.

## Abbreviations

The following abbreviations are used in this manuscript:

Ecal	Electromagnetic calorimeter
ECT	Straw-tube tracker
EMC	Electromagnetic calorimeter
FFD	Fast-forward detector
FHCal	Forward Hadron calorimeter
GS	Gas system
HMI	Human–machine interface
IT	Inner detector
ITS	Inner tracking system
JINR	Joint Institute for Nuclear Research
MPD	Multipurpose detector
MRPC	Multigap resistive plate chambers
NICA	Nuclotron-based Ion Collider fAcility
P&ID	Piping and instrumentation diagram
PID	Proportional-integral-derivative controller
PLC	Programmable logic controllers
SCADA	Supervisory control and data acquisition
SC Coil	Superconductor solenoid
ToF	Time-of-flight system
TPC	Time projection chamber
ZDC	Zero-degree calorimeter

## Appendix A

The P&ID drawing of the target gas system is shown in Figure A1.
